# Influence of Monometallic and Bimetallic Phytonanoparticles on Physiological Status of Mezquite

**DOI:** 10.1515/biol-2019-0008

**Published:** 2019-03-20

**Authors:** Daniel Gonzalez-Mendoza, Benjamín Valdez-Salas, Erick Bernardo-Mazariegos, Olivia Tzintzun-Camacho, Federico Gutiérrez-Miceli, Víctor Ruíz-Valdiviezo, Ludwi Rodríguez-Hernández, Gabriela Sanchez-Viveros

**Affiliations:** 1 Universidad Autonoma De Baja California, Mexicali, Mexico; 2 Instituto de Ingeniería de la Universidad Autónoma de Baja California, Calle de la Normal s/n y Boulevard Benito Juárez, 21100, Mexicali, Baja California, México; 3 Departamento de Ing. Química y Bioquímica, Tecnológico Nacional de México.Instituto Tecnológico de Tuxtla Gutiérrez, Tuxtla-Gutiérrez, Chiapas, México; 4 Instituto de Ciencias Agrícolas de la Universidad Autónoma de Baja California (ICA-UABC). Carretera a Delta s/n C.P. 21705, Ejido Nuevo León, Baja California, México; 5 Tecnológico Nacional de México/Instituto Tecnológico Superior de Cintalapa. Carretera Panamericana Km 995, C.P. 30400. Cintalapa, Chiapas, México; 6 Universidad Veracruzana. Facultad de Ciencias Agrícolas. Circuito Gonzalo Aguirre Beltrán s/n. Universidad Veracruzana. Xalapa, Veracruz, México

**Keywords:** *Justicia spicigera*, phyto-nanoparticles, *Prosopis glandulosa*, chlorophyll fluorescence, agronanotechnology

## Abstract

The present study was conducted to evaluate the impact of monometallic and bimetallic nanoparticles (NPs) of copper (Cu) and silver (Ag) from *Justicia spicigera* on the photochemical efficiency and phenol pattern of *Prosopis glandulosa*. In this study, the existence of localized surface plasmon resonance absorption associated with the nano-sized nature of Ag, Cu and Cu/Ag particles was confirmed by the presence of a single peak around 487, 585, and 487/580 nm respectively. Zeta potential and electrophoretic mobility were found to be 0.2 mV and 0.02 μmcm/(Vs) for synthesized NPs indicating less stability and thus tendency to agglomerate, and broad distribution of particles. Cu-NPs and Cu/Ag-NPs demonstrate that the dispersed phase is stable and has a minimum particle size at zeta potentials above –30 mV. Changes in phenolic compounds, total chlorophyll, and photochemical efficiency in leaves exposed to Ag, Cu and Cu/Ag phyto-nanoparticles were evaluated up to 72 hours. The results revealed that Ag-NP and Cu-NP from *J. spicigera* at 100 mg/L showed significant reduction in chlorophyll, epidermal polyphenol content and photochemical efficiency of *P. glandulosa*. In contrast, the application of bimetallic Cu/Ag-NP from *J. spicigera* showed a positive impact on physiological parameters of *P. glandulosa* after 72 h of exposure.

## 1 Introduction

Recently, attention has been drawn to the use of plants as a source of bioactive compounds for reduction of metal-nanoparticles (metal-NP) for the elimination of harmful reagents and effective synthesis of expected products through an economical method [1, 2]. Though metal-NPs are found naturally, there should be no doubt that anthropogenic activities play a major role in environmental contamination by nanoparticles [3, 4]. Recent nanotoxicological studies show that Ag and Cu nanoparticles have the potential to cause negative effects on growth and transpiration rates on species of *Bacopa monnieri*, *Triticum aestivum*, *Phaseolus radiates*, respectively [5, 6, 7 , 8]. Additionally, the presence of Ag or Cu-NPs could cause physiological and biochemical changes that adversely affect growth and productivity by reducing photosynthesis in terms of the levels of chlorophyll and induction of excessive amounts of reactive oxygen species (ROS) in plants [9]. Due to its ecological and commercial importance, *Prosopis* species has been extensively used in recent years to study the effects of heavy metal contamination in the agroecosystem [10]. Even though the physiological response to heavy metals in *Prosopis* species has been previously studied, the impact of metal-based phyto-NP on photochemical efficiency and total phenolic content in leaves of *P. glandulosa* are scarce. Therefore, the aim of the present work was to evaluate the changes in phenolic compound accumulation and photochemical efficiency in leaves of *P. glandulosa* exposed to Ag, Cu (monometallic) and Cu/Ag (bimetallic) phytoNPs.

## 2 Methods

### 2.1 Biosynthesis of nanoparticles (NPs)

The different nanoparticles (Ag, Cu and Ag:Cu) used in the present study were previously obtained from fresh and healthy leaves of *J. spicigera* plants according to the method proposed by Bernardo-Mazariegos [11].

**Figure 1:**
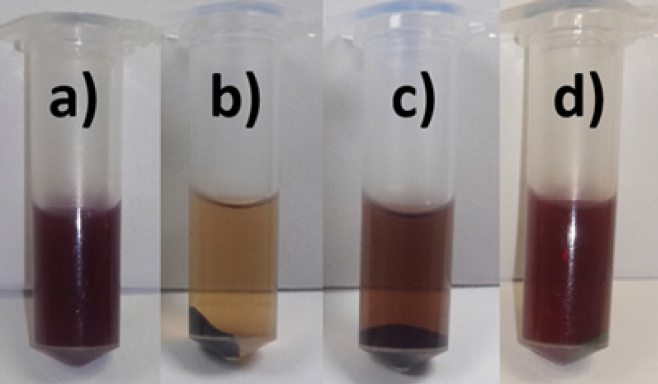
Green synthesis of metal-nanoparticles using extracts of *Justicia spicigera*: a) aqueous extract; b) Cu-phytonanoparticles; c) Ag-phytonanoparticles; d) Ag/Cu phytonanoparticles.

For metal-NP synthesis, 10 mL of aqueous *J. spicigera* leaf extract were added into 50 mL of aqueous solution of 10 mM silver nitrate, copper sulphate and silver nitrate/copper sulfate (1:1) for 15 min at 60°C, respectively. The bio-reduction of Cu, Ag and Cu/Ag (1:1) ions was observed by color change from yellow to brown, indicating the formation of NPs at room temperature (Figure 1). The AgNPs were purified by centrifugation at 11,200 *g* for 15 min and the precipitate was thoroughly washed with sterile distilled water to get rid of any unwanted impurities, and then transferred to a freeze dryer (the powder obtained was used in physiological assays).

### 2.2 Characterization of AgNPs

The process of bio-reduction of Cu, Ag and Cu/Ag (1:1) and the production of NPs, was determined using a spectrophotometer (DR6000™ UV VIS Spectrophotometer, USA) in the wavelength range 350–500 nm, with a resolution of 2 nm and using double distilled water as a blank reference.

### 2.3 Dynamic Light Scattering (DLS) and laser Doppler velocimetry (LDV)

DLS and LDV, for characterization of size and zeta potential of the phytonanoparticles in solution, were performed on a Nanotrac Wave instrument (Microtrac) according to the method proposed by Ruiz-Romero [12]. Measurements were made by means of Dynamic Light Scattering (DLS) in the range of 0.1-1000 μm at 25°C, using laser wavelength of 780 nm and a scattering angle of 90°. Finally, the DLS data obtained were analyzed using Microtrac FLEX operating software.

### 2.4 Determination of total phenol and flavonoid contents

The total phenol and flavonoid contents of the extract and metal-NP from *J. spicigera* were determined by the Folin-Ciocalteau and aluminum chloride colorimetric methods, according to Cervantes-Garcia [13].

The total phenol content was obtained from the calibration curve, and the results were expressed as mg of gallic acid equivalent per gram of dry extract (mg/g) at 760 nm against blank. The content of flavonoids was calculated as mg quercetin equivalents (QE) per gram of dry extract (mg/g). The calibration curve was prepared with quercetin solutions at concentrations from 10 to 100 mg mL^-1^ in methanol at 510 nm.

### 2.5 *Prosopis glandulosa* germination

*Prosopis glandulosa* seeds previously disinfected with ethanol 70% for 5 min were germinated and grown under laboratory conditions in plastic pots (four seeds per pot) filled with quartz sand and peat moss sterilized by autoclaving at 121°C for 2 h. The seedlings were grown for 4 weeks under 12 h light/12 darkness conditions with 60 % relative air humidity. The seedlings were irrigated daily with deionized water, and every other week were fertilized with Hoagland solution [14].

### 2.6 Nanoparticle exposure

One-month-old *P. glandulosa* plants (*n* = 4) were randomly collected and transferred to individual plastic containers holding 300 mL of water solution prepared with 100 ppm (according to previous studies from our laboratory) of AgNPs, CuNPs and Cu/Ag NPs (relation 1:1), respectively. Physiological parameters in plants were evaluated in each treatment group after 72 h under hydroponic conditions. Control plants for each group were transferred to plastic containers with 300 mL of water without NPs. These exposures were performed in quadruplicate.

### 2.7 Measuring of Chlorophyll fluorescence in *P. glandulosa* leaves

The determination of chlorophyll fluorescence was carried out using a portable fluorometer (OS-30p, OPTI-SCIENCE, USA) on completely expanded leaves. The data were recorded from 10 ms up to 1 s with data acquisition at every 10 ms for the first 300 ms, then every 100 ms up to 3 ms and every 1 ms thereafter. The signal resolution was 12 bits (0–4,000). For each treatment, the chlorophyll (Chl) a fluorescence transients of 4 individual leaves were measured at 24, 48 and 72 h after exposure to metal-NP. Leaves were maintained in darkness for 10 min before taking the data on chlorophyll fluorescence. The maximal intensity of the light source, providing an irradiance saturating pulse of 3,000 mmol photons.m^−2^.s^−1^ was used according to Gonzalez-Mendoza [15]. The ratio of variable fluorescence to maximal fluorescence (Fv/Fm) is an indicator of the efficiency of the photosynthetic apparatus and was calculated according to the method of Küpper [16].

### 2.8 Determination of leaf chlorophyll and Epidermal polyphenol content

To measure of leaf chlorophyll **(**Chl) and polyphenol content (EPhen), the adaxial side of the leaf was measured at 24, 48 and 72 h after exposure to metal-NP using the Dualex (FORCE-A, Orsay, France) and were expressed in arbitrary units (a.u.) according to Gonzalez-Mendoza [15].

### 2.9 Statistical analysis

Differences between the treatments were analyzed with one-way analysis of variance (ANOVA) and means were compared using Tukey’s test (p≤0.05), using SAS Version 9.0.

Significant differences were accepted if *p* ≤ 0.05 and data was expressed as mean ± standard error. Four biological replicates were analyzed for each variable (chlorophyll fluorescence, leaf chlorophyll and Epidermal polyphenol content).

## 3 Results

**Figure 2:**
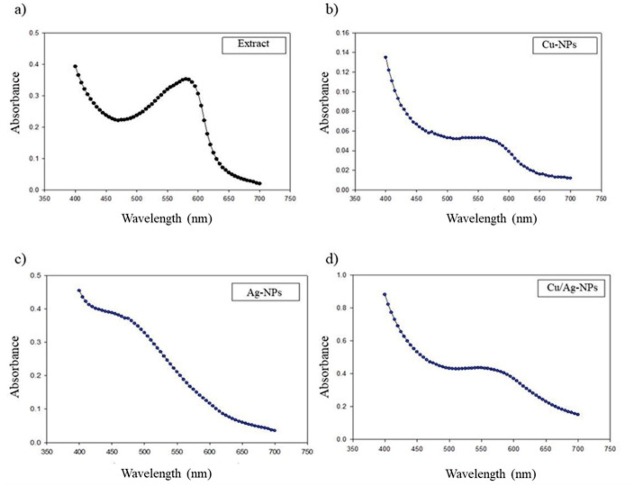
UV-Vis absortion spectrum of phytonanoparticles from *Justicia spicigera* extract: a) *Justicia spicigera* extract; b) CuNPs ; c) AgNPs; d) Cu-AgNPs

**Figure 3:**
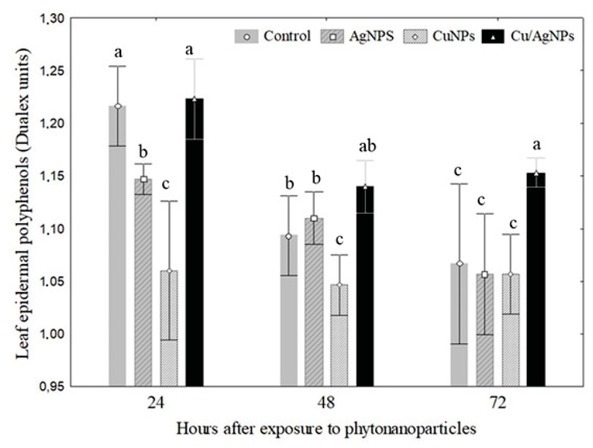
Epidermal polyphenols (Dualex units) in the leaves of *Prosopis glandulosa* exposed to phytonanoparticles from *Justicia spicigera* extract during an exposure period of 72 h. Different letters above bars indicate significant differences (one way ANOVA, post-ANOVA Tukey’s test).

UV-Vis absorption spectra have been proved to be quite sensitive to the formation of metal-nanoparticle colloids (Fig. 1) because nanoparticles exhibit an intense absorption peak due to the surface plasmon. In this study we observed the UV spectra of metal colloids in the range of 300 to 700 nm. Well-defined plasmon bands were observed around 487, 585, and 487/580 nm for Ag, Cu and Cu/Ag, respectively (Fig. 2). The peak of each metal-NP at different absorbance is due to the particle density, which is strongly associated with nano-sized nature of Ag, Cu and Cu/Ag particles. The DLS results for particle size in solution for the Ag, Cu and Cu/Ag nanoparticles are presented in Table 1. Our results showed that Ag-NPs from *J.spicigera* tended to form agglomerates of similar size when dispersed in water. Additionally, zeta potential and electrophoretic mobility were found to be 0.2 mV and 0.02 μmcm/(Vs) for synthesized AgNPs indicating less stability and thus tendency to agglomerate, and broad distribution of particles. On the other hand, Cu-NP and Cu/Ag-NP from *J.spicigera* exhibited a different pattern by agglomerating, 225.3 and 53.3 nm, respectively, when dispersed in water. Zeta potential varied widely for these samples, Cu-NPs and Cu/Ag-NPs in aqueous solutions demonstrate that the dispersed phase is stable and has a minimum particle size at zeta potentials above –30 mV (Table 1). In the present study statistical data revealed that the different metal-nanoparticles from *J. spicigera* differentially affect the content of EPhen in *P. glandulosa* leaves (Fig. 3). The results showed that in Cu/Ag-NPs treated plants, EPhen content increased significantly (*p*< 0.05) after 48h and 72 h exposure.

**Table 1:** DLS and LDV data for NP from *Justicia spicigera*

Particle*	DLS	LDV	

	Average diameter (nm)	Zeta potential ζ (mV)	Electrophoretic mobility U (μmcm/(Vs))
*AgNPs*	190.4	0.2	0.02	
*CuNPs*	225.3	-98	7.66	
*Cu/AgNPs*	53.3	-0.2	0.02	

*Particles were dispersed in H_2_O

**Figure 4:**
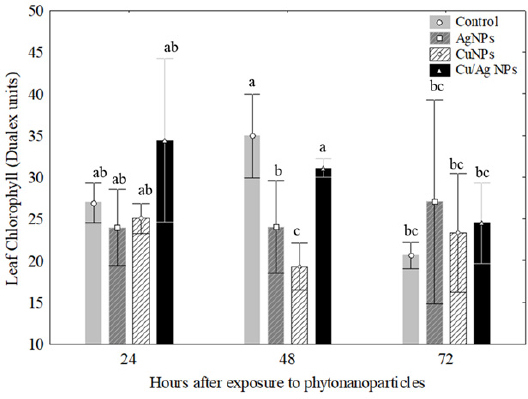
Changes in leaf chlorophyll (Dualex units) in the leaves of *Prosopis glandulosa* exposed to phytonanoparticles from *Justicia spicigera* extract during an exposure period of 72 h. Different letters above bars indicate significant differences (one way ANOVA, post-ANOVA Tukey’s test).

Otherwise a tendency for decreased EPhen values was found in plants in response to Cu-NPs with respect to control during 24h and 72 h of exposure. Finally, the *P. glandulosa* treated with Ag-NPs did vary with time and doses employed in this experiment (Fig. 3). In the present study *P. glandulosa* treated with 100 ppm of AgNPs, CuNPs and Cu/Ag NPs from *J. spicigera*, respectively, showed non significant changes in the Chl values compared to control during 24 h and 72 h of exposure (Fig. 4). However, significant differences for the Cu/Ag-NP treatment were registered for Chl in *P.glandulosa* after 48 hours of exposure compared with plants treated with Ag and Cu nanoparticles (Fig. 4).

**Figure 5:**
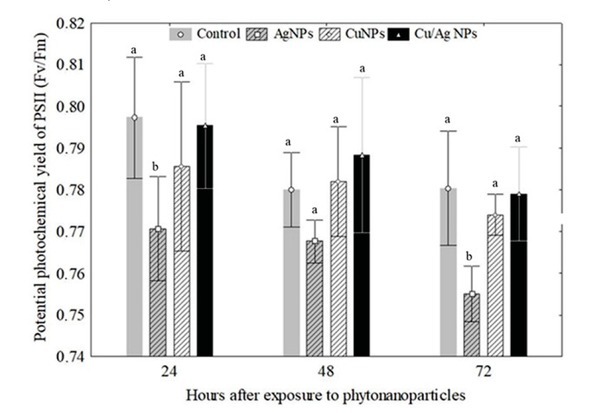
Variations in potential photochemical yield of PSII in the leaves of *Prosopis glandulosa* exposed to phytonanoparticles from *Justicia spicigera* extract during an exposure period of 72 h. Different letters above bars indicate significant differences (one way ANOVA, post-ANOVA Tukey’s test).

As shown in Table 2, total phenol and flavonoid contents of the aqueous extract and metal-NP from *J. spicigera* differed significantly. For example, Cu-NP (219.25 mg GAE g^− 1^) had the highest total phenol content, followed by Ag-NP (163.13 mg GAE g^− 1^), Cu/Ag-NP (108.69 GAE g^− 1^) and leaf extract of *J. spicigera* (71.49 mg GAE g^− 1^). On the other hand, leaf extract of *J.spicigera* had the highest total flavonoid content, followed by Cu-NP, Cu/Ag-NP and Ag-NP (Table 2). The measurements of chlorophyll a fluorescence showed a significant decrease (*p*< 0.05) in the photochemical efficiency (Fv/Fm) value of *P. glandulosa* treated with AgNPs from *J.spicigera* after 72 hours of exposure compared to control (Fig. 5). In plants treated with Cu and Cu/Ag-NPs, Fv/Fm activity did not show significant change with respect to control plants (Fig.5).

**Table 2:** Total phenolic and flavonoid content in extract and NPs from *Justicia spicigera*

Metal-nanoparticles	Total phenolic content ( mg GAE/g)	Total flavonoid content ( mg QE/g)
J. *spicigera* (control)	71.49±0.50^c^	2.21±0.01^a^
Ag-NPS	163.13±2.05^b^	1.83±0.04 ^c^
Cu-NPs	219.25±2.94^a^	2.10±0.07^b^
Cu/Ag-NPs	108.69±3.05^ab^	1.95±0.14^ab^

Results are expressed as mean **±** standard deviation of values from triplicate experiments. Values with the same letter (a, or b) within each column are equal according to Tukey`s test at *p*≤0.05.

## 4 Discussion

In this study the synthesis of Cu or Ag and Cu/Ag nanoparticles from *J.spicigera* showed brown to reddish color in aqueous solution (Fig. 1). Ruiz-Romero [2] and Keihan [17] reported that Ag and Cu nanoparticles from fresh *Yucca shilerifera* and green tea leaves, respectively, exhibited striking colors, from light yellow to brown in aqueous solution due to the increasing of synthesis of metallic nanoparticles formed as a result of reduction of Ag and Cu ions by secondary metabolites present in the aqueous solution of these plant extracts. In this case, the

flavonoids and phenols compounds in the leaf extract of *J.spicigera* could be responsible of the reduction of Ag, Cu and Cu/Ag to metal-nanoparticles and provision of stability. As previously reported, [18] metal ions form intermediate complexes with different functional groups, particularly the phenolic compounds present in plant extract. These complexes consequently reduce metal (*eg*., silver and copper) ions and act as capping agents. However, the probable mechanism is unclear and further investigations are required. Several studies have shown that metal-nanoparticles can have positive and negative effects on the physiological status of plants depending upon the properties of nanomaterials and the mode of application in different plant species [19]. The results of the present study showed that the reduction in Fv/Fm values in *P. glandulosa* could be a result of negative effects of AgNPs in the structure and composition of photosystem II reaction centers (PS II-RCs) according to Navarro [20]. A similar result has been reported by Jiang [21] who showed that AgNPs significantly decreased physiological parameters such as chlorophyll and chlorophyll fluorescence in *Spirodela polyrhiza*. In this study, Cu-NPs at a concentration of 100 ppm showed significant reduction in leaf chlorophyll and leaf epidermal polyphenol content of *P. glandulosa*, specifically during the first hours of treatment (24 and 48 h). Similar results were reported by Nair [22] and Costa [23], respectively, who observed that the application of Cu-nanoparticles (2 to 50 ppm) showed significant reduction in total chlorophyll content and inhibited the growth of plants. Another study showed that treatment of *Elodea densa* with Cu nanoparticles (1 mg/L) significantly reduced the content of photosynthetic pigments and chlorophyll fluorescence [24]. In contrast, the application of bimetallic Cu-Ag-NPs from *J. spicigera* showed a positive impact on physiological parameters of *P. glandulosa* after 72 h of exposure. According to previous work [25], the bimetallic nanoparticles have drawn a greater interest than the monometallic nanoparticles from a biotechnological point of view. In our study Cu/Cu bimetallic NP have more significant effect and showed higher increase in leaf epidermal polyphenols and this may be due to the synergistic effect arising from the Cu/Ag bimetallic NPs. According to Mazhar et al. [26], synergistic effects of two metals in bimetallic nanoparticles can enable certain functions which are otherwise not possible with monometallic nanoparticles alone. Finally bimetallic nanoparticles using plant material provide great opportunities in the field of agriculture and their understanding is important for the effective use of this nanoparticle in various aspects of agronanotechnology. Understanding some positive effects of phytonanoparticles in plants is of vital importance to the fields of agriculture and forestry.

## 5 Conclusion

The present study showed that bimetallic Cu-Ag-NPs (100 ppm) from *Justicia spicigera* have a positive impact on physiological parameters of *Prosopis glandulosa*. Further evaluations in the field are necessary to elucidate fully the biotechnological potential of *Justicia spicigera* Cu-AgNPs.
